# Neutralizing Antibodies Responses against SARS-CoV-2 in a Sardinian Cohort Group Up to 9 Months after BNT162b2 Vaccination

**DOI:** 10.3390/vaccines10040531

**Published:** 2022-03-29

**Authors:** Giuseppina Sanna, Alessandra Marongiu, Davide Firinu, Cristina Piras, Gianluigi Franci, Massimiliano Galdiero, Giuseppe Pala, Vanessa Palmas, Fabrizio Angius, Roberto Littera, Andrea Perra, Germano Orrù, Marcello Campagna, Giulia Costanzo, Federico Meloni, Ferdinando Coghe, Luchino Chessa, Aldo Manzin

**Affiliations:** 1 Microbiology and Virology Unit, Department of Biomedical Sciences, University of Cagliari, 09042 Monserrato, Italy; alemarongiu91@hotmail.it (A.M.); g.pala29@studenti.unica.it (G.P.); vanessapalmas@hotmail.it (V.P.); fangius@unica.it (F.A.); aldomanzin@unica.it (A.M.); 2 Department of Biomedical Sciences, University of Sassari, 07100 Sassari, Italy; 3 Department of Medical Sciences and Public Health, University of Cagliari, 09042 Monserrato, Italy; orru@unica.it (G.O.); mcampagna@unica.it (M.C.); g.costanzo@aoucagliari.it (G.C.); fe.meloni@aoucagliari.it (F.M.); luchinochessa@unica.it (L.C.); 4 Clinical Metabolomics Unit, Department of Biomedical Sciences, University of Cagliari, 09042 Monserrato, Italy; cristina.piras@unica.it; 5 Department of Medicine, Surgery and Dentistry “Scuola Medica Salernitana”, University of Salerno, 84081 Baronissi, Italy; gfranci@unisa.it; 6 Department of Experimental Medicine, University of Campania Luigi Vanvitelli, 80138 Naples, Italy; massimiliano.galdiero@unicampania.it; 7 Department of Internal Medical Sciences, Medical Genetics, “R. Binaghi” Hospital, University of Cagliari, 09126 Cagliari, Italy; roberto.littera@atssardegna.it; 8 Associazione per l’Avanzamento della Ricerca per i Trapianti O.d.V., Non Profit Organisation, 09100 Cagliari, Italy; 9 Oncology and Molecular Pathology Unit, Department of Biomedical Sciences, University of Cagliari, 09100 Cagliari, Italy; andrea.perra@unica.it; 10 Laboratory of Clinical Chemical Analysis and Microbiology, University Hospital of Cagliari, 09042 Monserrato, Italy; fcoghe@aoucagliari.it

**Keywords:** COVID-19, BNT162b2 vaccine, CLIA, Antibody, neutralization assay

## Abstract

Severe acute respiratory syndrome-related coronavirus 2 (SARS-CoV-2), the etiological agent of COVID-19, has caused over 460 million cases of infection and over 6 million deaths worldwide. The pandemic has called for science, technology, and innovation to provide solutions and, due to an incredible scientific and financial global effort, several prophylactic and therapeutic apparatuses such as monoclonal antibodies and vaccines were developed in less than one year to address this emergency. After SARS-CoV-2 infection, serum neutralizing antibodies are produced by B cells and studies on virus-neutralizing antibodies’ kinetics are pivotal. The process of protective immunity and the duration of this kind of protection against COVID-19 remain to be clarified. We tested 136 sera from 3 groups of individuals, some of them providing multiple sequential sera (1—healthy, no previous CoV2-infected, vaccinated; 2—healthy, previous CoV2 infected, vaccinated; 3—healed, previous CoV2-infected, not vaccinated) to assess the kinetics of antibodies (Abs) neutralizing activity. We found that SARS-CoV-2 infection elicits moderate neutralizing antibody activity in most individuals; neither age nor gender appear to have any influence on Abs responses. The BNT162b2 vaccine, when administered in two doses, induces high antibodies titre endowed with potent neutralizing activity against bare SARS-CoV-2 in in vitro neutralizing assay. The residual neutralization capability and the kinetic of waning immunity were also evaluated over 9 months after the second dose in a reference group of subjects. Neutralization titre showed a decline in all subjects and the median level of S-protein IgG, over 270 days after the second vaccination dose, was below 10 AU/mL in 53% of serum tested.

## 1. Introduction

Severe acute respiratory syndrome coronavirus 2 (SARS-CoV-2) was identified for the first time in Wuhan, Hubei province of China at the end of December 2019 [1]. On 7 January 2020, a new virus was isolated and identified by real-time reverse transcription polymerase chain reaction (RT-PCR) and next-generation sequencing (NGS) techniques: not even a decade after the Severe Acute Respiratory Syndrome (SARS) epidemic, another coronavirus (CoV), highly related to bats’ SARS-like viruses, was posing a new unimaginable challenge [2,3]. A few days later, Zhang et al. sequenced and published the viral genome on GenBank (MN908947) and more public platforms (https://www.ncbi.nlm.nih.gov/nuccore/MN908947 accessed on 20 February 2022), already marking the start of an era of massive international efforts to face the new threat. Already defined as a Public Health Emergency of International Concern (PHEIC) [4], the infection was officially declared a pandemic by the WHO on 11 March 2020 [5]. The first case of COVID-19 identified in Italy was isolated in Codogno [6] in February 2020 and from there, various waves of the pandemic followed one another until the approval of vaccines. In less than a year, effective vaccines were developed. Many of those currently approved by The European Medicines Agency (EMA) provide for the inoculation of two doses after a set time to enhance the antibody response [7,8].

The main target of vaccines was established to be the SARS-CoV-2 spike protein (S-protein), a large class I trimeric fusion protein which plays a pivotal role in viral pathogenesis [9]. The European Medicines Agency (EMA) approved BNT162b2, produced by Pfizer-BioNTech, as one of the first vaccines (ema.europa.eu). This vaccine contains mRNA translating for SARS-CoV-2 spike protein wrapped in lipid nanoparticles and its use in Italy was approved by AIFA Agency (Agenzia Italiana del Farmaco) at the end of December 2020. To date, 133,123,458 doses of vaccine have been dispensed in Italy. In Sardinia, 1,282,820 people are fully (two doses) vaccinated (salute.gov.it). In our study, we aim to retrospectively analyze and correlate antibody (Ab) responses and the neutralization capability induced by a double dose vaccination course with BNT162b2 in a cohort of 136 Sardinian subjects, which comprised both previously SARS-CoV-2 infected and naive subjects. Moreover, the specific IgG immune response and neutralizing Ab profile of a patient subgroup (*n* = 15) was analyzed during the long period (over 270 days) after the second dose. During the development of this study, guidelines regarding the administration of a single or double dose in subjects who had previously been infected by SARS-CoV-2 were still conflicting [10,11]. Since the vaccination campaign has started, various studies have shown that the measurements of Ab titre and the neutralizing capacity of Abs produced after the vaccine in previously infected subjects was much higher than the one developed by naive subjects [12,13,14]. In fact, in previously infected subjects an Ab titre equal to that of naive subjects after two doses was obtained after a single dose [15,16]. In this research, subjects were evaluated for serum SARS-CoV-2 Ab IgG to RDB with chemiluminescent immunoassays (CLIA) and microneutralization assay (MNA) at the day of second dose and 30 days after the second inoculum of BNT162b2 to investigate the Ab levels in previous natural infection and infection-naïve subjects, and to assess further correlations with age, gender or co-morbidities. Several studies have shown the persistence, after more than six months, of Ab titre induced by the second dose of BNT162b2 [17,18]. However, recent literature has reported different dynamics of Abs decrease [19]; therefore, vaccine-induced antibody kinetics may be non-homologous in all individuals [20,21]. Starting from this consideration, the residual neutralization capability and the kinetic of immunity were also evaluated more than 270 days after the second dose in a reference group of subjects.

## 2. Materials and Methods

Cohort size. The present study enrolled a cohort of Regione Sardinia participants (*n* = 136) from 2 groups of individuals: the first of healthy, not previously CoV-2 infected, and vaccinated subjects; the second of healthy, previously CoV-2 infected, and vaccinated patients. For all participants three serum samples were collected between January and October, 2021; the samples were taken on the day of the second dose of Pfizer-BioNTech BNT162b2 vaccine, thirty days after, and, for a selected group of patients, also over 270 days after the second inoculum. Blood samples belong to subjects enrolled in the study protocol at the Teaching Hospital of the Cagliari University, Department of Biomedical Sciences and Department of Medical Sciences and Public Health, University of Cagliari. A total of 136 patients were enrolled (45/136 males, 33% and 91/136 females, 67%), with median age of 45. The aliquots of serum sample were collected at said hospital and stored at −80 °C for the following analysis. The previous natural infection was evaluated by a positive rt-PCR test for SARS-CoV-2 through nasopharyngeal swab test.

Patients were recruited and enrolled in the study protocol at the University Hospital of Cagliari (Italy). Written informed consent was collected from all patients and controls in accordance with the ethical standards (institutional and national) of the local human research committee. The study protocol, including informed consent procedures, conforms to the ethical guidelines of the Declaration of Helsinki and was approved by the Ethics Committee of the Cagliari University Hospital on 27 May 2020; protocol number GT/2020/10894 and extension approved 27 January 2021. 

SARS-CoV-2 Antibody Immunoassays. This assay was performed according to the previously published method [22] with slight modifications. Briefly, three time points were evaluated at the day of booster (Time I), after 30 (Time II) and over 270 (Time III)days from the second dose of BNT162b2. SARS-CoV-2 S-RBD IgG CLIA for the in vitro quantitative determination of antibodies (IgG) against spike Receptor Binding domain in human serum was performed on Maglumi 800 analyzer (SNIBE—Shenzhen New Industries Biomedical Engineering Co., Ltd., Shenzhen, Cina). The results were expressed in arbitrary units (AU) per millilitre and, according to the manufacturer’s claims, a test result ≥ 1.10 AU/mL was considered positive.

SARS-CoV-2 Microneutralization assay. MNA was performed in Biosafety Level 3 (BSL-3) laboratory (Section of Microbiology and Virology, Cittadella Universitaria di Monserrato). Serum samples were diluted (1:2; 1:5; 1:10; 1:40; 1:160; 1:640) in triplicates and mixed with 100 TCID_50_ of SARS-CoV-2 virus (clinical isolate, strain VR PV10734, kindly donated by the Lazzaro Spallanzani Hospital of Rome, Italy) at 37 °C; serum/virus mixes were transferred to 96-well plates containing 5 × 10^5^/mL adherent Vero E6 (ATCC, Manassas, Virginia, United States) cells seeded in the previous day. Plates were incubated at 37 °C for 72 h prior evaluation of CPE via microscope and were then fixed and stained with Gram’s crystal violet solution. The neutralization percentage of individual dilutions was calculated by setting the mean OD_595_ of the serum control equal to 100%. Virus dilution used for infection was titrated in each experiment. Cell growth and serum controls were run in each experiment. Neutralization titres of serum samples were determined by the highest serum dilution protecting 90% of the infected wells [23].

### Statistical Analysis

GraphPad Prism software (version 7.01, GraphPad Software, Inc., San Diego, CA, USA) was used to perform the univariate statistical analysis. The collected dataset was subjected to the Shapiro–Wilk univariate normality test [24] in order to evaluate the normality of the data. Due to the absence of normal distribution, nonparametric testing was performed using Kruskal–Wallis tests. In the different scatter plot, the central horizontal line shows the median concentration, and the error bars show the 25th and 75th percentiles (interquartile range (IQR), 25–75th percentile), respectively. *p*-value < 0.05 was considered a statistically significant difference between medians.

## 3. Results

Data on a total of 136 subjects (24 previously infected and 112 not infected) were collected retrospectively, after gathering information about the period of infection, the development of symptoms during said period, and the subjects’ activity as healthcare workers in the hospital or lack thereof (Table 1). Among the 136 individuals, 45 (33%) were males and 91 (67%) females, with an average age of 46.7 for males and 44.47 for females. All subjects were further interviewed to obtain data on comorbidities. Those affecting a low number of individuals, and therefore having a marginal impact on Anti S-RBD Ab and neutralization titres, have not been reported.

Among 112 SARS-CoV-2 naive subjects,24 individuals (21.4%) had one or more comorbidities (12 had cardiovascular diseases without association with diabetes (*n* = 4), 2 had past or present cancer, 24 had immuno-related diseases). Within our cohort, 88 subjects worked in a hospital (64.6%). The infected and vaccinated subjects were all infected at the time of the first wave in Italy, before the start of the vaccination campaign in Italy, so most were infected with the original SARS-CoV-2 strain and a small part by lineage B.1.1.7. Participants’ characteristics are displayed in Table 1.

### 3.1. Antibody Dynamics

On the day of the second vaccine dose, we found a mild elevation of anti S-RBD IgG median levels in serum samples of naive subjects, measured by Maglumi 800 analyzer (CLIA method) (Table 2), with median IgG levels of 35.02 AU/mL (14.22–70.27). As expected, elevated anti S-RBD IgG median levels were found in samples from previously infected patients: 856.8 AU/mL(506.55–1000). No relevant differences in age and sex were found at this time point in IgG levels.

Among previous naturally infected patients, the median level of S-protein IgG 30 days after the second dose of BNT162b2 vaccine was higher: 706.45 UA/mL(157.97–983.2), or greater >1000 UA/mL in 8/24 subjects (33%) in comparison to antibody levels in patients without prior COVID-19 history: 195.7 UA/mL (99.20–349.67). Indeed, second dose vaccines elicited a strong immune response in 97% of naive subjects (only 3/112 subjects resulted as not responders). This result is an expected finding, as the vaccine worked as a booster of naturally occurring immunity, in agreement with published data [25,26].

The titres determined in male and female subjects not previously infected 30 days after the second dose revealed an increasing trend compared to the measurements of Ab titre determined after the day of the second dose (Table 2).

No significant anti-S-RBD level differences were found between males and females in all the study conditions (Table 2).

### 3.2. Neutralization Titres

The development of neutralizing antibodies is correlated to protection and a description of the kinetics profile of SARS-CoV-2 neutralizing responses is pivotal. Several studies have shown antibody responses in vaccinated and COVID-19 patients using ELISA, pseudoparticles assay, or similar binding assays that might not completely reflect neutralization to the same extent as the neutralization assays employing live SARS-CoV-2. In this study, we determined the 90% endpoint by microneutralization assay in order to assess the serum neutralization titre; this assay is more stringent than its 50% equivalent, which is generally employed in current research.

**Figure 1:**
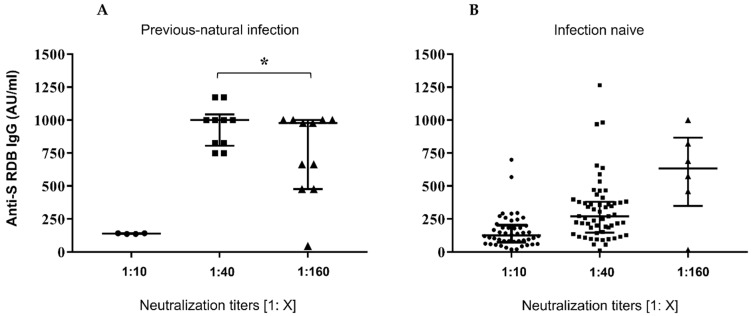
Serological responses after two doses of BNT162b2 mRNA vaccine. (**A**,**B**) Comparative anti S-RBD Abs and neutralization titres measured at 30 days after the second inoculum in COVID-19 infection-naive and previousnatural infection subjects. Distribution of variables was evaluated by Shapiro–Wilk test. Non-parametric variables were expressed as median (IR, interquartile range). We compared IgG Ab levels with in vitro virus neutralization by using one-way ANOVA Kruskal–Wallis tests (*p* < 0.0001) * *p* = ns.

Figure 1, panel A, reports the dot-plots of anti-S-RBD levels in previously infected subjects vs. neutralization titres, while panel B shows anti-S-RBD levels in samples from patients without prior COVID-19 experience.

**Figure 2:**
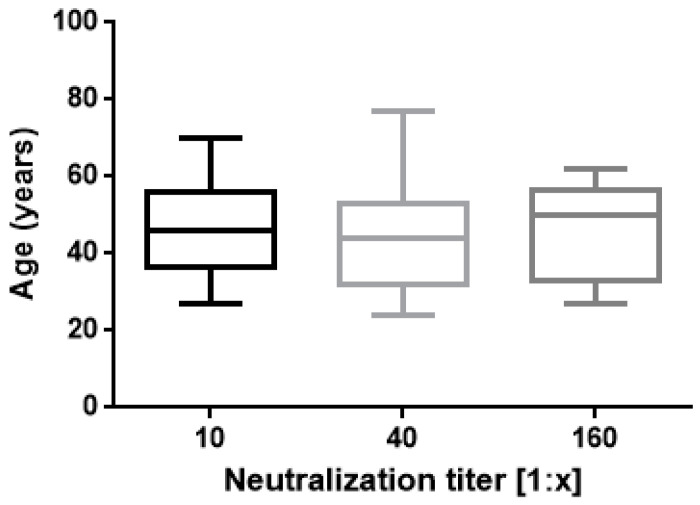
Neutralization titres 30 days after second vaccination are shown. Boxes span the interquartile range; the line within each box denotes the median and whiskers indicate the 25 and 75 percentile values (one way ANOVA, *p* = 0.5 ns).

The results of this study confirm proved differences in vaccine responses between infection-naive subjects and ones with a history of natural infection. A strong correlation was found between IgG Ab levels and neutralizing activity (Figure 1) as previously reported by Padoan and coworkers [27]. In regard to neutralization titres and age, no significant correlation was found (Figure 2).

**Figure 3:**
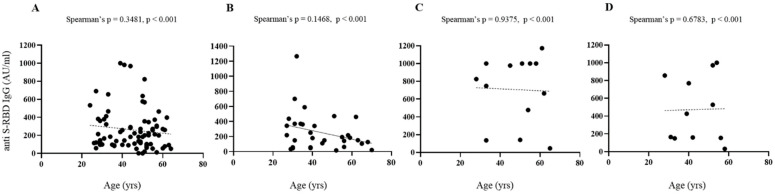
Serological responses after two doses of BNT162b2 mRNA vaccine. (**A**–**D**) Spearman’s correlation of post vaccine anti-S RBD antibodies levels and age, gender in infection-naive ((**A**), females; (**B**), males) and previously infected ((**C**), females; (**D**), males) subjects with superimposed linear regression lines (95% confidence interval).

Figure 3 shows comparative levels of anti-SARS-CoV-2 S-RBD IgG in infection-naive and previously infected subjects. Considering anti S-RBD levels, age and sex, no significant differences were found in all conditions considered.

The influence of gender, age and smoke on the trend of these results deserves further study. In fact, the number of participants who have been previously subjected to a natural infection is significantly lower than that of naive subjects; therefore, the first category is not adequately represented in all the age groups taken into consideration for this study.

**Figure 4:**
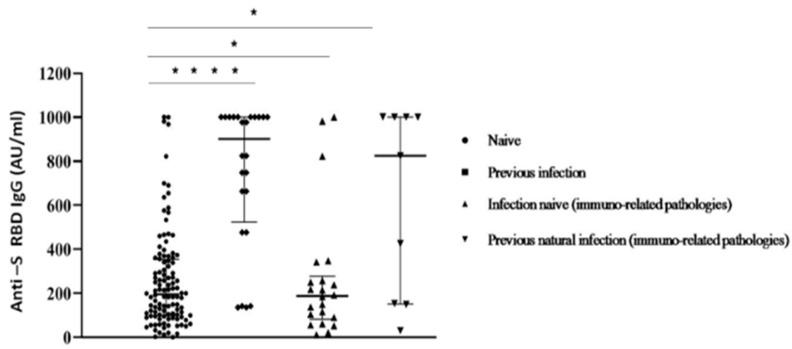
Anti S-RBD Ab titres measured at 30 days after the second inoculum in COVID-19 infection-naive and previous natural infection subjects with/without immuno-related diseases. Each symbol represents a single study participant. Bars represent means (one way ANOVA, (* *p* < 0.05; **** *p* < 0.0001)).

Despite this limitation, our data correspond with other recent studies [14,27]. When comparing participants afflicted by immuno-mediated diseases with healthy subjects among the whole group, significant differences in Ab levels were reported (Figure 4).

The COVID-19 pandemic was still ongoing through the duration of our study, and understanding the extent and kinetics of the protective immunity determined by the second vaccine dose became a critical issue. Such knowledge was required to endorse a booster dose and to prioritize its inoculum in vulnerable patients.

We selected a subgroup of 15 COVID-19 naive subjects from the initial group of 136 participants to analyze the trend of neutralizing activity and anti-SARS-CoV-2 S-IgG Ab levels after more than nine months after the second BNT162b2 dose (median time from dose 1 to dose 2 was 21 days). 

**Figure 5:**
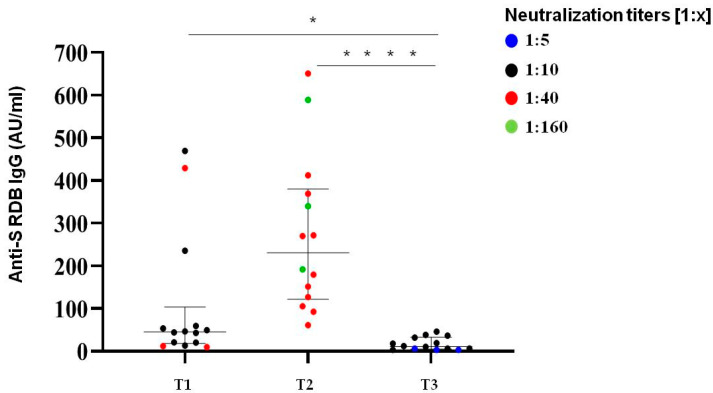
Immunological responses over 270 days after two doses of BNT162b2 mRNA vaccine. Comparative anti S-RBD Abs and neutralization titres measured at T1 (time of second dose), T2 (30 days after second dose) and T3 (>270 days after the second inoculum) in COVID-19 infection-naive subjects. Distribution of variables was evaluated by Shapiro–Wilk test. Non-parametric variables were expressed as median (IR, interquartile range). We compared IgG Ab levels with in vitro virus neutralization using one-way ANOVA Kruskal–Wallis tests (* *p* < 0.05; **** *p* < 0.0001).

Neutralization titres showed a decline in all subjects; however, the majority (80%) of the samples still retained a detectable neutralization capacity on the threshold of 1:10 (Figure 5). Thus, the neutralization titres over 270 days after the first two doses of mRNA vaccine were comparable to those detected at the time of second dose. Only 3/15 subjects exhibited low neutralization titres (1:5) below the established limit of positivity.

Longitudinal studies have previously reported a continuous increase in the frequency of Spike+ and Spike+ RBD+ memory B cells from 3 to 6 months post-vaccination in SARS-CoV-2 naïve individuals, as a consequence of an increase in the quality and breadth of the immune response in germinal centres [28,29].

The residual neutralizing activity shown about 9 months after mRNA vaccination, coupled with anamnestic B-cell response and T-cell response published data [30], is in line with the observed reduction of protection from symptomatic infection associated with a persistence of significant protection from severe COVID-19 symptoms [31].

Among SARS-CoV-2 naive patients, the median level of S-protein IgG over 270 days (T3) after the second vaccination dose was about 11 UA/mL (5.88–28.7) in male and female patients, which confirmed the same kinetic behaviour highlighted with neutralization titres. A total of 53% of analyzed subjects (8/15) had Ab levels below 10 AU/mL.

As this study originated after preliminary screening, some data are lacking compared to other screenings in the literature [32,33,34]; however, our results are in line with those obtained by other authors [13,20,35,36]. A recent study on the Qatar population [37] has analyzed the trend in protection induced by BNT162b2 against new infections.

A peak is observed in the first month after the second dose and then a gradual decrease month after month before reaching a minimum level five to seven months after the second dose.

The decline accelerated after the fourth month, and effectiveness reached approximately 20% of the initial value in months 5 to 7 after the second dose. Instead, the effectiveness against hospitalization and death did not decline over time, except possibly in the seventh month after the second dose.

The emergence of SARS-CoV-2 variants has enhanced concerns about the width of neutralizing Ab responses that should be continuously monitored after infection and vaccination. The kinetics observed in our study group seems to correspond with that described before the arise of variants of concern. 

A significant waning of the humoral response, within 6 months of the second dose, was observed in different cohorts and at this time is critical to evaluate anti-S-RBD Abs and neutralization titres after a third BNT162b2 booster. Several recent studies have found that a third BNT162b2 dose in healthcare workers [38] and aged 60 years and older [39] was associated with significantly increased IgG titres and no major adverse effects. This investigation is also ongoing for our study cohort.

We are aware that our study had limitations. Firstly, the analyzed cohort is small if compared to most clinical cohorts and our results may be attributable to patient selection. Secondly, considering the limited number of clinical specimens, we were not able to test the neutralization activity against new arising variants of concern. Undoubtedly, it is important to know how current variants affect the efficacy of available vaccines and how neutralization titres are correlated to protection.

However, employing live SARS-CoV2 virus in neutralisation assays instead of a surrogate model further strengthens this study. In addition, the 90% neutralisation titre endpoint used in our study is more stringent than the 50% commonly reported in most published studies. 

Moreover, our cohort included individuals subjected to a high risk of infection, such as healthcare workers and patients with co-morbidities, therefore not limiting the range to healthy subjects or those with a low risk of infection.

## 4. Conclusions

This research shows data on vaccination responses consistent with recent literature and highlights the need of monitoring neutralizing Ab responses to also define the time-point at which titres turn undetectable. As they are mostly produced by short-lived plasmablasts, Abs will naturally and considerably decline over time, both after natural infection and vaccination. Consequently, differences between induced levels of memory B cells, under different conditions (i.e., following infection, or in the absence of the infection but following vaccination), will be responsible for the reappearance of circulating neutralizing antibodies after natural infection or third dose. Furthermore, the presence of neutralizing antibodies could only partially explain the protection against serious disease. Due to the lack of sterilizing immunity (like in SARS-CoV-2), an important role could indeed be played by T lymphocytes, both in the management of airway infection and in the severity of the disease.

In this scenario, further research is required and T-cell responses should be analyzed to better understand immune responses after the third vaccination dose in our study groups.

## Figures and Tables

**Table 1:** Cohort participant characteristics.

	PreviouslyInfected Subjects (*n* = 24)	Naive Subjects (*n* = 112)
Age (years)	46.37	44.47
Gender		
Male (%)	8.1%	25%
Female (%)	9.5%	57.3%
Healthcare workers (%)	12.3%	52.3%
Others (%)	3.8%	31.5%
Smokers	8.3%	8%
Diabetes	4%	3.6%
Cardiovascular diseases	-	10.7%
Immuno-related diseases	37%	21.4%
Disease severity (%)		
Asymptomatic	33.3%	-
Mild	29.2%	-
Severe	29.2%	-
Unknown	8%	-

Disease severity: Asymptomatic = 0 days of symptoms, mild = 7–15 days of symptoms, severe ≥ 20 days of symptoms, unknown = lack of data.

**Table 2:** Vaccine immune response of cohort subjects.

	Time I Median AU/mL (25th and 75th Percentile)		Time II Median AU/mL (25th and 75 th Percentile)	
	Male	Female	Male	Female
Previously infected	607.9 (215.67–971.07)	1000 (761.65–1000)	426.6 (156.65–813.25)	825 (477.1–1000)
Not previously infected	46.52 (13.97–79.15)	33.87 (14.49–66.05)	181.85 (88.96–356.7)	200.8 (100.69–341.27)

Time I = anti SARS-CoV-2 median measurements of Ab titre (expressed as AU/mL) at the time of second dose of BNT162b2 vaccine; Time II = anti SARS-CoV-2 median measurements of Ab titre (expressed as AU/mL) 30 days after the second dose of BNT162b2 vaccine.

## Data Availability

Data and materials are available from corresponding authors on reasonable request.
